# Supplementary material for: Icotinib, Almonertinib, and Olmutinib: A 2D Similarity/Docking-Based Study to Predict the Potential Binding Modes and Interactions into EGFR
Source: Molecules. 2021 Oct 24;26(21):6423. doi: 10.3390/molecules26216423 (PMC8588130; doi:10.3390/molecules26216423)
Supplement: Supplementary file 1 [file molecules-26-06423-s001.zip › molecules-1420195-supplementary.pdf]

Table S1: Similarity scores (MCS Tanimoto) of icotinib, almonertinib, and olmutinib against the EGFR co-crystallized ligand database

No	Pdb/name	Compound (smiles)	MCS Tanimoto with		
			Icotinib	Almonertinib	Olmutinib
1.	Icotinib	C#CC1=CC(=CC=C1)NC2=NC=NC3=CC4=C(C=C32)OCCOCCOCCO4	1.0000	0.1724	0.1852
2.	Almonertinib	CN(C)CCN(C)C1=CC(=C(C=C1NC(=O)C=C)NC2=NC=CC(=N2)C3=CN(C4=CC=CC=C43)C5CC5)OC	0.1724	1.0000	0.3455
3.	Olmutinib	CN1CCN(CC1)C2=CC=C(C=C2)NC3=NC4=C(C(=N3)OC5=CC=CC(=C5)NC(=O)C=C)SC=C4	0.1852	0.3455	1.0000
4.	0UN	CN1CCN(CC1)c2ccc(c(c2)OC)Nc3ncc(c(n3)Oc4cccc(c4)NC(=O)C=C)Cl	0.1852	0.3962	0.8421
5.	3IKA	CN1CCN(CC1)c2ccc(c(c2)OC)Nc3ncc(c(n3)Oc4cccc(c4)NC(=O)C=C)Cl	0.1852	0.3962	0.8421
6.	5HG5	CCC(=O)Nc1cccc(c1)Oc2c3cc[nH]c3nc(n2)Nc4ccc(cc4)N5CCN(CC5)C	0.2549	0.3455	0.7949
7.	5HG8	CCC(=O)Nc1cccc(c1)Oc2c3cc[nH]c3nc(n2)Nc4cnn(c4)C	0.2128	0.1964	0.5000
8.	5X26	CC(C)n1c2c(cnc(n2)Nc3ccc(cc3)N4CCN(CC4)C)nc1Nc5ccccc5	0.2400	0.3585	0.4167
9.	5X27	CN1CCN(CC1)c2ccc(cc2)Nc3ncc4c(n3)n(c(n4)Nc5ccccc5)C6CCCC6	0.2308	0.3455	0.4000
10.	5GMP	CC1=CC(=O)N(c2c1cnc(n2)Nc3ccc(cc3OC)N4CCN(CC4)C)c5cccc(c5)NC(=O)C=C	0.2593	0.3684	0.3962
11.	5D11	Cc1cc(n[nH]1)Nc2cc(nc(n2)Oc3cccc(c3)NC(=O)C=C)N4CCN(CC4)C	0.2200	0.1833	0.3958
12.	5X28	CN1CCN(CC1)c2ccc(cc2)Nc3ncc4c(n3)n(c(n4)Nc5ccccc5)C6CCCCC6	0.2264	0.3393	0.3922
13.	5UWD	CCC(=O)Nc1cccc(c1)Nc2c(cnc(n2)Nc3ccc(cc3OC)N4CCN(CC4)C(=O)C)C(F)(F)F	0.2321	0.3621	0.3636
14.	5XDK	CC(=O)N1CCN(CC1)c2ccc(c(c2)OC)Nc3ncc(c(n3)Nc4cccc(c4)NC(=O)C=C)C(F)(F)F	0.2321	0.3621	0.3636
15.	4ZAU	CN1C=C(C2=CC=CC=C21)C3=NC(=NC=C3)NC4=C(C=C(C(=C4)NC(=O)C=C)N(C)CCN(C)C)OC	0.1786	0.9487	0.3585
16.	6LUD	Cn1cc(c2c1cccc2)c3ccnc(n3)Nc4cc(c(cc4OC)N(C)CCN(C)C)NC(=O)C=C	0.1786	0.9487	0.3585
17.	6Z4B	CCC(=O)Nc1cc(c(cc1N(C)CCN(C)C)OC)Nc2nccc(n2)c3cn(c4c3cccc4)C	0.1786	0.9000	0.3585
18.	6JRK	CCC(=O)N1CCCC(C1)N2c3c(cnc(n3)Nc4ccc(c(c4)C)N5CCN(CC5)C)C(=C(C2=O)c6cccc(c6Cl)F)C	0.2542	0.2923	0.3559
19.	5GTZ	CCN1CCN(CC1)c2cc(c(cc2NC(=O)C=C)Nc3ncc(c(n3)Nc4ccccc4S(=O)(=O)C(C)C)Cl)OC	0.2241	0.4727	0.3509
20.	6JRX	CCC(=O)NC1CCC(CC1)N2c3c(cnc(n3)Nc4ccc(c(c4)C)N5CCN(CC5)C)CN(C2=O)c6ccccc6Cl	0.1774	0.2969	0.3390
21.	4QQC	CCC(=O)Nc1ccc(cc1)CN2c3c(cnc(n3)Nc4ccc(cc4)N5CCN(CC5)C)CN(C2=O)c6cc(cc(c6)OC)OC	0.1515	0.2836	0.3226
22.	5UGA	CC(C)n1cnc2c1nc(nc2Nc3ccc(cc3)N4CCN(CC4)C)NC5CCN(C5)C(=O)C	0.2549	0.2759	0.3208
23.	7AEM	CN1CCN(CC1)C2CCN(CC2)c3ccc(c(c3)OC)Nc4ncc(c(n4)Nc5ccccc5P(=O)(C)C)Cl	0.2321	0.3167	0.3158
24.	5Y9T	CCc1c(nc(c(n1)C(=O)N)Nc2ccc(cc2)N3CCC(CC3)N4CCN(CC4)C)OC5CCN(C5)C(=O)C=C	0.1864	0.2308	0.3103
25.	7AEI	Cn1cc(cn1)c2cc(c(cc2N3CCC(CC3)N(C)C)OC)Nc4ncc(c(n4)Nc5ccccc5P(=O)(C)C)Cl	0.2241	0.3065	0.3051
26.	3LZB	c1ccc(cc1)CC(=O)Nc2cccc(c2)c3c(n4ccsc4n3)c5ccnc(n5)Nc6ccc(cc6)N7CCOCC7	0.1613	0.3443	0.3000
27.	5EM6	CN1CCN(CC1)c2ccc(cc2)NC(=O)C3=C(C=CNC3=O)Nc4ccnc(n4)N	0.2500	0.2500	0.2941
28.	5EM7	CN1CCN(CC1)c2ccc(cc2)NC(=O)C3=C(C=CNC3=O)Nc4ccccc4OC	0.2449	0.2456	0.2885
29.	5EM5	CN1CCN(CC1)c2ccc(cc2)NC(=O)C3=C(C=CNC3=O)NCCc4ccc(cc4)Cl	0.1481	0.2414	0.2830
30.	4R5Y	c1cc2c(cc1C(F)(F)F)nc([nH]2)C3C4C3Oc5c4cc(cc5)Oc6ccnc7c6CCC(=O)N7	0.1636	0.1562	0.2727
31.	6DI3	C=CC(=O)NC1CCN(C1)c2ccc(c(n2)Oc3ccc(cc3)Oc4ccccc4)C(=O)N	0.1698	0.2203	0.2593
32.	4R5S	CCC(=O)Nc1ccc(cc1)CN(c2cc(ncn2)Nc3ccc(cc3)N4CCN(CC4)C)C(=O)Nc5c(c(cc(c5Cl)OC)OC)Cl	0.2031	0.2254	0.2576
33.	6DI5	CCC(=O)N1CC2CC1CN2c3ccc(c(n3)Oc4ccc(cc4)Oc5ccccc5)C(=O)N	0.1667	0.2167	0.2545

**34.**	**4LI5**	CCC(=O)Nc1ccc(c(c1)Nc2ncc(c(n2)c3c[nH]c4c3cccc4)Cl)OC	0.2041	0.6829	0.2500
**35.**	**3W2S**	c1cc(cc(c1)Oc2ccc(cc2Cl)Nc3c4c(ccn4CCOCCO)ncn3)NC(=O)NC5CCCCC5	0.2778	0.1449	0.2500
**36.**	**7A2A**	CCC(=O)Nc1cccc(c1)Nc2c(cnc(n2)Nc3ccc(cc3)OCCOC)F	0.2766	0.2500	0.2453
**37.**	**6HVE**	COc1c2c(c[nH]c2ncn1)c3cccc(c3)NC(=O)C=C	0.2143	0.2200	0.2391
**38.**	**2HWO**	C=CC(=O)Nc1ccc2c(c1)c(ncn2)Nc3ccccc3	0.5000	0.2200	0.2391
**39.**	**4LQM**	C=CC(=O)Nc1ccc2c(c1)c(ncn2)Nc3cccc(c3)Br	0.4857	0.2157	0.2340
**40.**	**5HG7**	CCC(=O)N1CC(C(C1)OC)COc2c3c(c[nH]c3nc(n2)Nc4cnn(c4)C)Cl	0.2041	0.1695	0.2264
**41.**	**6HVF**	CCN1C=Nc2c(c(c([nH]2)c3ccc(cc3)N4CCN(CC4)C)c5cccc(c5)NC(=O)C=C)C1=O	0.1607	0.4727	0.2241
**42.**	**5HG9**	CCC(=O)N1CC(C(C1)C(F)(F)F)COc2c3cc[nH]c3nc(n2)Nc4cnn(c4)C	0.2000	0.1667	0.2222
**43.**	**6S89**	CCC(=O)Nc1cccc(c1)c2c3c([nH]c2c4ccc(cc4)N5CCN(CC5)C)ncnc3OC(C)C	0.1579	0.1875	0.2203
**44.**	**5HI2**	CNC(=O)c1cc(ccn1)Oc2ccc(cc2)NC(=O)Nc3ccc(c(c3)C(F)(F)F)Cl	0.1731	0.1833	0.2182
**45.**	**6MNY**	c1cc(ccc1c2c(c(n(n2)C3CCCN(C3)C#N)N)C(=O)N)Oc4ccc(cc4F)F	0.1509	0.1452	0.2182
**46.**	**6S8A**	CCC(=O)Nc1cccc(c1)c2c3c([nH]c2c4ccc(cc4)N5CCN(CC5)C)ncnc3OCC(C)C	0.1552	0.1846	0.2167
**47.**	**2QQ7**	CN(C)CC=CC(=O)Nc1ccc2c(c1)c(ncn2)Nc3cccc(c3)Br	0.4359	0.2000	0.2157
**48.**	**5D12**	C=CC(=O)Nc1ccc2c(c1)nc(nc2Nc3cc[nH]n3)c4ccccc4	0.3333	0.2453	0.2157
**49.**	**6DI9**	CC(C)(C)NC(=O)c1ccc(cc1)Nc2c(ccc(n2)N3CCC(C3)NC(=O)C=C)C(=O)N	0.2400	0.2000	0.2143
**50.**	**5YU9**	C=CC(=O)N1CCCC(C1)n2c3c(c(n2)c4ccc(cc4)Oc5ccccc5)c(ncn3)N	0.1481	0.1613	0.2143
**51.**	**6VHN**	CCC(=O)Nc1ccc(c(c1)Nc2cc(ccn2)c3c(nc([nH]3)SC)c4ccc(cc4)F)OC	0.2353	0.4314	0.2105
**52.**	**3PP0**	c1cc(cc(c1)Oc2cc(cc(cn2)Nc3c4c(ccn4CCOCCO)ncn3)Cl)C(F)(F)F	0.2857	0.1406	0.2105
**53.**	**4LRM**	CCC(=O)Nc1ccc2c(c1)c(ncn2)Nc3cccc(c3)Br	0.4857	0.1923	0.2083
**54.**	**6J6M**	CCC(=O)N1CCC(CC1)C2CCNc3n2nc(c3C(=O)N)c4ccc(cc4)Oc5ccccc5	0.1429	0.1385	0.2069
**55.**	**3W33**	c1cc(c2ccsc2c1)Oc3ccc(cc3Cl)Nc4c5c(ncn4)NCCC(=C5)C(=O)NCCO	0.2549	0.1562	0.2069
**56.**	**5Y25**	c1cc(c(cc1Nc2c3cc(c(cc3ncn2)OCCCN4CCOCC4)NC(=O)C5CCCN5C(=O)CF)Cl)F	0.4000	0.1940	0.2063
**57.**	**6V6O**	CCC(=O)Nc1ccc(c(c1)Nc2cc(ccn2)c3c(nc([nH]3)CCCO)c4ccc(cc4)F)OC	0.2264	0.4151	0.2034
**58.**	**6V66**	CCC(=O)Nc1ccc(c(c1)Nc2cc(ccn2)c3c(nc([nH]3)SCCOC)c4ccc(cc4)F)OC	0.2222	0.4074	0.2000
**59.**	**3POZ**	CC(C)(CC(=O)NCCn1ccc2c1c(ncn2)Nc3ccc(c(c3)Cl)Oc4cccc(c4)C(F)(F)F)O	0.2885	0.1493	0.1967
**60.**	**3W32**	CS(=O)(=O)CCNC(=O)C1=Cc2c(ncnc2Nc3ccc(c(c3)Cl)Oc4cccc(c4)C(F)(F)F)NCC1	0.2364	0.1471	0.1935
**61.**	**3W2P**	CN(C)CCCC(=O)NCCn1ccc2c1c(ncn2)Nc3ccc(c(c3)Cl)Oc4cccc(c4)C(F)(F)F	0.2830	0.1471	0.1935
**62.**	**6DUK**	c1cc(ccc1c2ccc3c(c2)C(=O)N(C3)C(c4cc(ccc4O)F)C(=O)Nc5nccs5)N6CCNCC6	0.1333	0.1642	0.1935
**63.**	**4I24**	COc1cc2c(cc1NC(=O)C=CCN3CCCCC3)c(ncn2)Nc4ccc(c(c4)Cl)F	0.4419	0.1803	0.1930
**64.**	**4I23**	COc1cc2c(cc1NC(=O)C=CCN3CCCCC3)c(ncn2)Nc4ccc(c(c4)Cl)F	0.4419	0.1803	0.1930
**65.**	**4G5J**	CN(C)CC=CC(=O)Nc1cc2c(cc1OC3CCOC3)ncnc2Nc4ccc(c(c4)Cl)F	0.5366	0.1774	0.1897
**66.**	**5JEB**	COc1ccc(cc1Nc2c3c(n[nH]c3ncn2)c4ccco4)c5ccco5	0.3571	0.2182	0.1887
**67.**	**2R4B**	CCc1cc2c(s1)c(ncn2)Nc3ccc(c(c3)Cl)OCc4cccc(c4)F	0.3571	0.1754	0.1887
**68.**	**6LUB**	CC(C)n1c2cc(cc(c2c3c1cc(nc3)Nc4ccnc(n4)c5cnn(c5)S(=O)(=O)C6CC6)OCC(F)(F)F)N7CCN(CC7)C	0.2459	0.1944	0.1884
**69.**	**1M17**	COCCOC1=C(C=C2C(=C1)C(=NC=N2)NC3=CC=CC(=C3)C#C)OCCOC	0.9333	0.1724	0.1852

**70.**	**4HJO**	COCCOc1cc2c(cc1OCCOC)ncnc2Nc3cccc(c3)C#C	0.9333	0.1724	0.1852
**71.**	**4RJ3**	COC1CCN(CC1)c2nccc(n2)Nc3cc4c(ccn4C5CCCC5)cn3	0.2609	0.1724	0.1852
**72.**	**4RJ5**	COC1CCN(CC1)c2nccc(n2)Nc3cc4c(cc([nH]4)c5c[nH]nc5)cn3	0.2609	0.1525	0.1852
**73.**	**3BEL**	COCCON=Cc1c(ncnc1Nc2ccc3c(c2)cnn3Cc4cccc(c4)F)N	0.2708	0.1639	0.1754
**74.**	**4WD5**	CCC(=O)Nc1cc(ccc1C)N2c3c4cc(ccc4ncc3C=CC2=O)c5c[nH]nc5	0.1731	0.1833	0.1754
**75.**	**4RJ4**	CC(C)n1c2cc(ncc2cc1c3c[nH]nc3)Nc4ccnc(n4)N5CCC(CC5)OC	0.2449	0.1639	0.1754
**76.**	**2RGP**	c1cc(cc(c1)F)Cn2c3ccc(cc3cn2)Nc4c(c(ncn4)N)CNN5CCCCC5	0.2653	0.1613	0.1724
**77.**	**6JZ0**	CN(C)CC=CC(=O)Nc1cccc(c1)c2c3c(ncnc3oc2c4ccccc4)NC(CO)c5ccccc5	0.1695	0.1618	0.1719
**78.**	**5EDR**	CC1(c2c(c(nc(n2)c3ccnn3C)Nc4c5ccccc5[nH]n4)CO1)C	0.3023	0.2453	0.1698
**79.**	**5EDQ**	CC1(c2c(c(nc(n2)c3c[nH]nc3)Nc4c5cccc(c5[nH]n4)Cl)CO1)C	0.3023	0.2453	0.1698
**80.**	**4G5P**	CN(C)CCCC(=O)Nc1cc2c(cc1OC3CCOC3)ncnc2Nc4ccc(c(c4)Cl)F	0.5366	0.1774	0.1695
**81.**	**5HID**	Cc1ccc(cc1Nc2ccc3c(c2)C(=O)N(C=N3)C)NC(=O)c4cccc(c4)C(C)(C)C#N	0.2600	0.1967	0.1695
**82.**	**6WA2**	CC(=O)Nc1cc(ccn1)c2c(nc([nH]2)SC)c3cccc(c3)NC(=O)c4cc(ccc4F)O	0.1667	0.1774	0.1695
**83.**	**5CAL**	Cc1nc2cnc(cc2n1C(C)C)Nc3ccnc(n3)NCC(C)(C)C(=O)N	0.2667	0.1552	0.1667
**84.**	**4JR3**	CC(=O)Nc1cccc(c1)c2c3c(ncnc3oc2c4ccccc4)NC(CO)c5ccccc5	0.1852	0.1562	0.1667
**85.**	**6D8E**	CCC(=O)Nc1cccc(c1)N(c2ccnc(n2)Nc3ccn(n3)C)C(=O)OCc4ccc(cc4)F	0.2500	0.2295	0.1639
**86.**	**5C8K**	COC1CCN(CC1)c2nccc(n2)Nc3cc4c(cn3)ncn4C5CCCC5	0.2609	0.1525	0.1636
**87.**	**4RJ6**	COC1CCN(CC1)c2nccc(n2)Nc3cc4c(cn3)[nH]c(n4)c5c[nH]nc5	0.2609	0.1525	0.1636
**88.**	**5CAO**	Cc1nc2cnc(cc2n1C(C)C)Nc3ccnc(n3)NCC(C)(C)S(=O)(=O)C	0.2609	0.1525	0.1636
**89.**	**5CAN**	Cc1nc2cnc(cc2n1C(C)C)Nc3ccnc(n3)N4CCC(C4)(C)C(=O)N	0.2609	0.1525	0.1636
**90.**	**5CAQ**	Cc1nc2cnc(cc2n1C(C)C)Nc3ccnc(n3)N4CCC(C(C4)F)OC	0.2609	0.1525	0.1636
**91.**	**6S9C**	CC(C)(Cn1c2ccccc2nc1NC(=O)c3ccnc(c3)c4ccccc4)O	0.1600	0.1724	0.1636
**92.**	**3K47**	CC(=Cc1coc2c1c(nc(n2)N)N)c3ccccc3OC	0.2143	0.1509	0.1633
**93.**	**6TFU**	CCC(=O)Nc1cccc(c1)c2c[nH]c3c2c(ncn3)Nc4ccc5c(c4)cnn5Cc6ccccc6	0.2941	0.1515	0.1613
**94.**	**5C8M**	Cc1nc2cnc(cc2n1C(C)C)Nc3ccnc(n3)N4CCC(CC4)S(=O)(=O)C	0.2553	0.1500	0.1607
**95.**	**4RJ7**	Cc1cc(nc(n1)NC(C)CO)Nc2cc(ccn2)NC(=O)c3c(cccc3Cl)Cl	0.2553	0.2321	0.1607
**96.**	**5HIC**	CC(C)n1cnc2c1cc(nc2)Nc3ccnc(n3)c4cnn(c4)S(=O)(=O)C5CC5	0.2553	0.2321	0.1607
**97.**	**6S9B**	CC(C)(Cn1c2ccccc2nc1NC(=O)c3cc(cc(c3)F)c4cccnc4)O	0.1569	0.1695	0.1607
**98.**	**6S9D**	c1ccc2c(c1)-c3cc(ccn3)C(=O)Nc4nc5ccccc5n4CCCCCO2	0.1800	0.1695	0.1607
**99.**	**3UG2**	COc1cc2c(cc1OCCCN3CCOCC3)C(=Nc4ccc(c(c4)Cl)F)N=CN2	0.3333	0.1290	0.1579
**100.**	**5C8N**	Cc1nc2cnc(cc2n1C(C)C)Nc3ccnc(n3)N4CCC(CC4)(CCN)OC	0.2500	0.1475	0.1579
**101.**	**5HCX**	Cc1nc2cnc(cc2n1C(C)C)Nc3ccnc(n3)c4cnn(c4)S(=O)(=O)C5CC5	0.2500	0.2281	0.1579
**102.**	**4JQ8**	CN(C)CCC(=O)Nc1cccc(c1)c2c3c(ncnc3oc2c4ccccc4)NC(CO)c5ccccc5	0.1724	0.1471	0.1562
**103.**	**4V0G**	CCC(=O)Nc1cccc(c1)n2c3ccccc3nc2c4c(ncc(n4)c5cnn(c5)C6CCN(CC6)C)N	0.1333	0.1642	0.1562
**104.**	**5HCY**	CC(C)n1cc(c2c1cc(nc2)Nc3ccnc(n3)c4cnn(c4)S(=O)(=O)C5CC5)C(=O)NC6CCOCC6	0.2364	0.2000	0.1562
**105.**	**1XKK**	CS(=O)(=O)CCNCc1ccc(o1)c2ccc3c(c2)c(ncn3)Nc4ccc(c(c4)Cl)OCc5cccc(c5)F	0.3269	0.1449	0.1538

**106.**	**4JRV**	CN(C)CCCC(=O)Nc1cccc(c1)c2c3c(ncnc3oc2c4ccccc4)NC(CO)c5ccccc5	0.1695	0.1449	0.1538
**107.**	**3W2Q**	CCOc1cc2c(cc1NC(=O)CCCN(C)C)c(c(cn2)C#N)Nc3ccc(c(c3)Cl)OCc4ccccn4	0.3800	0.1618	0.1538
**108.**	**6TFV**	CCC(=O)Nc1cc(ccc1OCCO)c2c[nH]c3c2c(ncn3)Nc4ccc(c(c4)Cl)OCc5ccccn5	0.2778	0.1449	0.1538
**109.**	**5J87**	Cc1c(cccc1NC(=O)c2ccc(cc2)C(C)(C)C)C3=CN(C(=O)C(=C3)Nc4ccc(c(c4)NC(=O)C=C)C(=O)N5CCOCC5)C	0.1846	0.1757	0.1528
**110.**	**6TFZ**	CCC(=O)Nc1cc(ccc1OCCO)c2c[nH]c3c2c(ncn3)Nc4ccc5c(c4)cnn5Cc6ccccc6	0.2727	0.1429	0.1515
**111.**	**6TFY**	CCC(=O)Nc1cc(ccc1OCCCO)c2c[nH]c3c2c(ncn3)Nc4ccc(c(c4)Cl)OCc5ccccn5	0.2727	0.1429	0.1515
**112.**	**5FED**	CCC(=O)N1CCCCC(C1)n2c3c(cccc3nc2NC(=O)c4cccc(c4)C(F)(F)F)C	0.1455	0.1587	0.1500
**113.**	**5CAU**	CC(c1nc2cnc(cc2n1C(C)C(F)(F)F)Nc3ccnc(n3)N4CCC(C(C4)F)OC)O	0.2353	0.1406	0.1500
**114.**	**6TFW**	CCC(=O)Nc1cc(ccc1OCCN(C)C)c2c[nH]c3c2c(ncn3)Nc4ccc(c(c4)Cl)OCc5ccccn5	0.2679	0.1408	0.1493
**115.**	**5FEQ**	Cc1cccc2c1n(c(n2)NC(=O)c3ccnc(c3)C)C4CCCCN(C4)C(=O)C=CCN(C)C	0.1429	0.1562	0.1475
**116.**	**6TG0**	CCC(=O)Nc1cc(ccc1OCCO)c2c[nH]c3c2c(ncn3)Nc4ccc(cc4)CN5C(=O)c6ccccc6C5=O	0.2632	0.1389	0.1471
**117.**	**6WAK**	CC(=O)Nc1cc(ccn1)c2c(nc([nH]2)SC)c3cccc(c3)NC(=O)c4ccccc4CN5Cc6ccccc6C5=O	0.1429	0.1549	0.1471
**118.**	**6TG1**	CCC(=O)Nc1cc(ccc1OCCN(C)C)c2c[nH]c3c2c(ncn3)Nc4ccc(cc4)CN5C(=O)c6ccccc6C5=O	0.2542	0.1351	0.1429
**119.**	**6WXN**	CC(=O)Nc1cc(ccn1)c2c(nc([nH]2)SC)c3cccc(c3)NC(=O)c4c(ccc(c4CN5C(=O)c6ccccc6C5=O)O)F	0.1364	0.1486	0.1408
**120.**	**6Z4D**	CCC(=O)NC1CN(CC1F)c2nc(c3c(n2)n(cn3)C)Nc4cn(nc4OC)C	0.2041	0.1500	0.1404
**121.**	**5UG8**	CCC(=O)NC1CN(CC1F)c2nc(c3c(n2)n(cn3)C(C)C)Nc4cnn(c4)C	0.2041	0.1311	0.1404
**122.**	**5HCZ**	CCC(C)n1c2cc(ncc2c(n1)N3CC(C3)C(C)(C)O)Nc4ccnc(n4)c5cnn(c5)S(=O)(=O)C6CC6	0.2143	0.2581	0.1385
**123.**	**3M11**	c1ccc(cc1)c2c3c(ncnc3oc2c4ccccc4)NCCc5ccc(cc5)NC(=O)Nc6ccccc6	0.1695	0.1449	0.1364
**124.**	**5UG9**	CCC(=O)NC1CN(CC1F)c2nc(c3c(n2)n(cn3)C(C)C)Nc4cn(nc4OC)C	0.1961	0.1452	0.1356
**125.**	**6P8Q**	c1ccc(cc1)CN2c3ccccc3Nc4ccc(cc4C2=O)F	0.2045	0.1887	0.1346
**126.**	**2JIU**	CCN1CCN(CC1)Cc2ccc(cc2)c3cc4c([nH]3)ncnc4NC(C)c5ccccc5	0.1923	0.1613	0.1333
**127.**	**4MHA**	CCCCNc1ncc(c(n1)NC2CCC(CC2)O)C(=O)NCc3ccc(cc3)S(=O)(=O)N	0.1481	0.1250	0.1333
**128.**	**6WU8**	CC1(CCN(CC1)c2cnc3c(n[nH]c3n2)c4cccc(c4Cl)Cl)N	0.1489	0.2308	0.1321
**129.**	**5D41**	c1ccc(cc1)C(C(=O)Nc2nccs2)N3Cc4ccccc4C3=O	0.1489	0.1228	0.1321
**130.**	**5GNK**	Cn1ccnc1COc2ccc(cc2Cl)c3c4c(ncnc4n(n3)C5CCCN(C5)C(=O)C=C)N	0.1636	0.1562	0.1290
**131.**	**5ZWJ**	c1ccc2c(c1)CN(C2=O)C(c3cc(ccc3O)F)C(=O)Nc4nccs4	0.1429	0.1186	0.1273
**132.**	**5GTY**	Cc1cccc(n1)COc2ccc(cc2Cl)c3c4c(ncnc4n(n3)C5CCCN(C5)C(=O)C=C)N	0.1607	0.1538	0.1270
**133.**	**5U8L**	C#CCNC(=O)c1cc(nc(n1)N2CCN(CC2)Cc3ccc(cc3)S(=O)(=O)F)Nc4cc(n[nH]4)C5CC5	0.1964	0.1324	0.1231
**134.**	**5XGN**	Cn1c2ccccc2c3c1c4c(c5ccccc5n4CCC#N)c6c3C(=O)NC6	0.1600	0.1930	0.1228
**135.**	**6LLX**	C(CC(=O)NC(CS)C(=O)NCC(=O)O)C(C(=O)O)N	0.0652	0.1132	0.1224
**136.**	**5J9Z**	Cn1cc(c2c1cccc2)c3c4c(ncnc4n(n3)C5CCCN(C5)C(=O)C=C)N	0.1569	0.2778	0.1207
**137.**	**6V5N**	c1ccc(cc1)c2cc3c(ccnc3[nH]2)c4c(nc([nH]4)CCCO)c5ccc(cc5)F	0.1321	0.1667	0.1186
**138.**	**4JQ7**	c1ccc(cc1)c2c3c(ncnc3oc2c4ccccc4)NC(CO)c5ccccc5	0.2000	0.1667	0.1186
**139.**	**6LLC**	Cc1c(c(no1)c2ccccc2)C(=O)Nc3ncc(s3)[N+](=O)[O-]	0.1556	0.1698	0.1154
**140.**	**6P8Q**	c1nc(c2c(n1)n(cn2)C3C(C(C(O3)COP(=O)(O)O)O)O)N	0.1556	0.1273	0.1154
**141.**	**2ITU**	CC12C(C(CC(O1)n3c4c(c5c3c6n2c7ccccc7c6c8c5C(=O)N=C8)CCCC4)NC)OC	0.1429	0.1935	0.1111

**142.**	**5HIE**	CC(C)(C)c1nc(c(s1)c2ccnc(n2)N)c3cccc(c3F)NS(=O)(=O)c4c(cccc4F)F	0.1429	0.1746	0.1111
**143.**	**6WVZ**	CC(=O)NC1C(C(C(OC1O)CO)O)O	0.1579	0.1020	0.1111
**144.**	**5HIB**	CC(C)(C)NC(=O)c1cnn2c1nc(cc2)NS(=O)(=O)c3ccnn3C	0.1458	0.1935	0.1091
**145.**	**4RIY**	c1nc(c2c(n1)n(cn2)C3C(C(C(O3)COP(=O)(O)OP(=O)(O)O)O)O)N	0.1429	0.1186	0.1071
**146.**	**5J9Y**	C=CC(=O)N1CCCC(C1)n2c3c(c(n2)c4cccc5c4cccc5)c(ncn3)N	0.1569	0.2321	0.1017
**147.**	**5D41**	c1nc(c2c(n1)n(cn2)C3C(C(C(O3)COP(=O)(O)OP(=O)(NP(=O)(O)O)O)O)O)N	0.1321	0.1111	0.1000
**148.**	**6V5P**	COCCCc1[nH]c(c(n1)c2ccc(cc2)F)c3ccnc4c3CC(=N4)c5ccccc5	0.1296	0.1639	0.0984
**149.**	**1CJ1**	c1cc(cc(c1)N)COC(=O)NC(Cc2ccc(cc2)OP(=O)(O)O)C(=O)NC3(CCCCC3)C(=O)NC4CCCCC4C(=O)N	0.1194	0.0897	0.0946
**150.**	**2GS6**	c1nc(c2c(n1)n(cn2)C3C(C(C(O3)COP(=O)(O)OP(=O)(O)OP(=O)(O)SCC(=O)N)O)O)N	0.1228	0.1045	0.0938
**151.**	**6LLC**	Cc1cc2c(cc1C)N(C3=NC(=O)NC(=O)C3=N2)CC(C(C(COP(=O)(O)OP(=O)(O)OCC4C(C(C(O4)n5cnc6c5ncnc6N)O)O)O)O)O	0.1233	0.1220	0.0864
**152.**	**6JKH**	c1cc(c[n+](c1)C2C(C(C(O2)COP(=O)([O-])OP(=O)(O)OCC3C(C(C(O3)n4cnc5c4ncnc5N)O)O)O)O)C(=O)N	0.1061	0.0921	0.0822
**153.**	**3K47**	c1nc(c2c(n1)n(cn2)C3C(C(C(O3)COP(=O)(O)OP(=O)(O)OCC4C(C(C(O4)N5C=CCC(=C5)C(=O)N)O)O)O)OP(=O)(O)O)N	0.1000	0.0875	0.0779
**154.**	**1X0N**	c1ccc2c(c1)cccc2CC3CC=CC(CC(=O)NC4(CCCCC4)C(=O)NC(C(=O)NC3)CC(=O)N)c5ccc(cc5)CP(=O)(O)O	0.0986	0.1139	0.0769
**155.**	**4X8Y**	Cc1c2n3c(c1CCC(=O)O)C=C4C(=C(C5=[N]4[Fe]36[N]7=C(C=C8N6C(=C5)C(=C8C)C=C)C(=C(C7=C2)C)C=C)C)CCC(=O)O	0.0746	0.0789	0.0685
**156.**	**6XL4**	CCC(=O)Nc1cc(c(cc1N(C)CCN(C)C)OC)Nc2nccc(n2)c3cn(c4c3cccc4)C	0.1786	0.9000	0.3585
**157.**	**6XL4**	c1ccc(cc1)CN2c3cc(ccc3Nc4ccccc4C2=O)F	0.2045	0.1887	0.1346

Calculated Molecular Properties of Icotinib *VS* Erlotinib

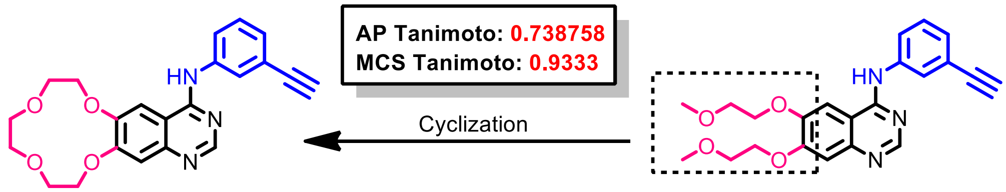

Icotinib

Molecular formula: C_{22} H_{21} N_3 O_4
Molecular weight: 391.15
Number of HBA: 6
Number of HBD: 1
MolLogP : 2.25
MolLogS : -2.78 (in Log(moles/L)) 654.65 (in mg/L)
MolPSA : 61.29 A2
MolVol : 383.05 A3
pKa of most Basic/Acidic group : 5.21 / 18.88
BBB Score : 3.80 The
Number of stereo centers: 0

Erlotinib (AQ4)

Molecular formula: C_{22} H_{23} N_3 O_4
Molecular weight: 393.17
Number of HBA: 6
Number of HBD: 1
MolLogP : 2.21
MolLogS : -2.73 (in Log(moles/L)) 735.34 (in mg/L)
MolPSA : 61.01 A2
MolVol : 388.72 A3
pKa of most Basic/Acidic group : 5.21 / 18.88
BBB Score : 3.81
Number of stereo centers: 0

Fig. S1. Structural relationship between icotinib and erlotinib and their calculated molecular properties.

Calculated Molecular Properties of Almonertinib *VS* Osimertinib

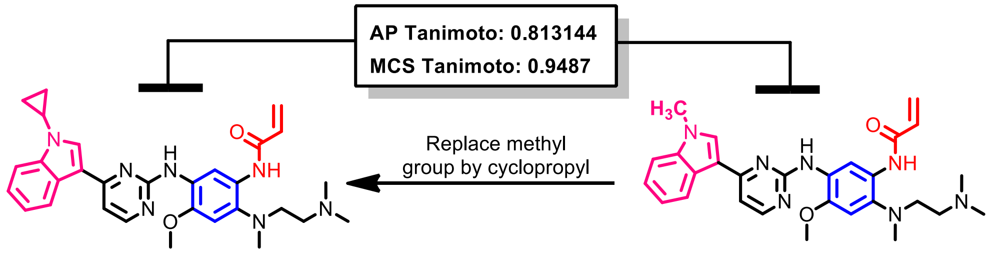

**Almonertinib**	**Osimertinib**
Molecular formula: $C_{30} H_{35} N_7 O_2$	Molecular formula: $C_{28} H_{33} N_7 O_2$
Molecular weight: 525.29 (> 500)	Molecular weight: 499.27
Number of HBA: 5	Number of HBA: 5
Number of HBD: 2	Number of HBD: 2
MolLogP : 4.81	MolLogP : 3.98
MolLogS : -4.54 (in Log(moles/L)) 15.03 (in mg/L)	MolLogS : -4.17 (in Log(moles/L)) 34.02 (in mg/L)
MolPSA : 67.53 A2	MolPSA : 67.73 A2
MolVol : 560.22 A3	MolVol : 524.71 A3
pKa of most Basic/Acidic group : 8.84 / 12.04	pKa of most Basic/Acidic group : 9.46 / 12.04
BBB Score : 3.12 (6-0)	BBB Score : 3.14 (6-0)
Number of stereo centers: 0	Number of stereo centers: 0

Fig. S2. Structural relationship between icotinib and erlotinib and their calculated molecular properties.

Calculated Molecular Properties of Olmutinib *VS* WZ4003

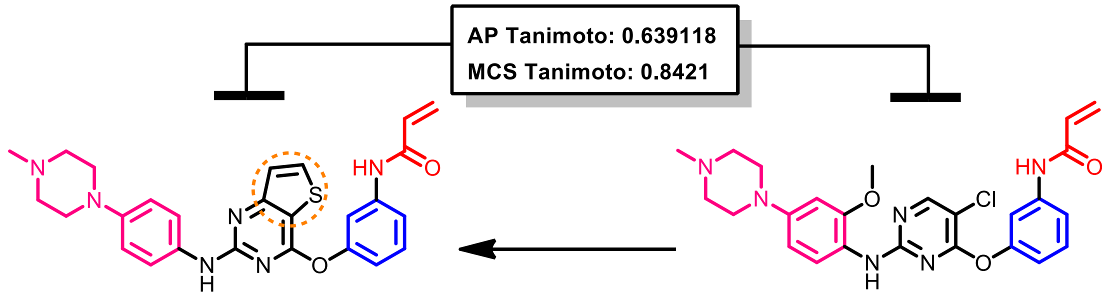

Olmutinib

Molecular formula: $C_{26}H_{26}N_6O_2S$
Molecular weight: 486.18
Number of HBA: 6
Number of HBD: 2
MolLogP : 4.97
MolLogS : -4.75 (in Log(moles/L)) 8.73 (in mg/L)
MolPSA : 67.17 A2
MolVol : 480.12 A3
pKa of most Basic/Acidic group : 7.76 / 13.62
BBB Score : 3.19 (6-0)
Number of stereo centers: 0

WZ4003

Molecular formula: $C_{25}H_{27}ClN_6O_3$
Molecular weight: 494.18
Number of HBA: 6
Number of HBD: 2
MolLogP : 4.18
MolLogS : -4.78 (in Log(moles/L)) 8.24 (in mg/L)
MolPSA : 73.89 A2
MolVol : 483.39 A3
pKa of most Basic/Acidic group : 7.76 / 13.62
BBB Score : 3.34 (6-0)
Number of stereo centers: 0

Fig. S3. Structural relationship between icotinib and erlotinib and their calculated molecular properties.
